# Behavior of Aspartic Proteases From Artichoke Flower (*Cynara cardunculus L. var Scolymus*) in the Hydrolysis of Buffalo Milk Casein

**DOI:** 10.1111/1750-3841.70773

**Published:** 2025-12-15

**Authors:** Rebeca Rodrigues Vieira Onelli, Josane Cardim de Jesus, Thinara de Freitas Oliveira, Leduan Rosa Alcântara, Renata Cristina Ferreira Bonomo, Leandro Soares Santos, Sibelli Passini Barbosa Ferrão

**Affiliations:** ^1^ Postgraduate Program in Engineering and Food Science (PPGECAL) State University of Southwest Bahia (UESB) Itapetinga Bahia Brazil; ^2^ Department of Food Science (DCA) Federal University of Lavras (UFLA) Lavras Minas Gerais Brazil; ^3^ Degree in State University of Southwest Bahia (UESB) Itapetinga Bahia Brazil

**Keywords:** chromatography, peptides, plant coagulant

## Abstract

The objective of this study was to assess the activity of proteases from artichoke flower extract in the enzymatic hydrolysis of casein from buffalo milk and to investigate its potential application in cheese production. Artichoke flower extract and microbial chymosin were assessed for their proteolytic activity (PA) and milk clotting activity (MCA) under varying conditions of pH, temperature, CaCl_2_, and NaCl concentration. The peptides obtained from the PA were analyzed using reverse phase high performance liquid chromatography, while the clots obtained from the MCA were analyzed through electrophoresis and mid‐infrared spectroscopy. Mini cheeses were produced under optimal conditions established by the specificity ratio (SR) value to evaluate the technological application of artichoke flower extract. The artichoke flower extract showed optimal PA conditions at pH 6.15 and temperature 45.2°C, and for MCA it showed maximum activity at pH 5.8 and temperature 56.9°C, values close to those found for chymosin. In the RP‐HPLC, SDS‐PAGE, and MIR, it was observed that the extract hydrolyzed casein in peptide bonds other than chymosin; however, its yield in cheese production remained unchanged. The artichoke flower has demonstrated potential as coagulant for buffalo cheese production, offering an alternative means to add economic value to these products.

## 1 Introducion

Buffalo milk production is the second largest in the world, contributing to 12.5% of global milk production and up to 15% of the global dairy market. Buffalo milk possesses distinct physical, chemical, and sensory properties, with higher levels of fat (7.5%), protein (4.1%), total solids (18%), and calcium (180 mg/100 mL) compared to cow's milk, which contains about 3.8% fat, 3.5% protein, and 120 mg/100 mL calcium. It also contains larger casein micelles compared to cow's milk (FAO 2019; Pantoja et al. 2022; Liao et al. 2025).

Buffalo milk, due to its nutritional, sensory, and technological characteristics, is an excellent alternative raw material for producing cheese, butter, yogurt, and other dairy products. The variety of products derived from this species milk has increased and filled supermarket shelves, however, the number of added value products is still low (Pantoja et al. 2022).

One way to increase the economic value of buffalo milk products is to create cheeses using plant‐based coagulants. These coagulants can enhance the flavor, aroma, and texture of the cheese. Some studies have already been carried out using vegetable coagulants for the production of cheese using milk from other species, such as cows (Jesus et al. 2023), goat (Liburdi et al. 2019) and sheep (Galán et al. 2008). Although there is also research involving buffalo milk, most of it focuses on the technological aspects of coagulation and cheese making. However, there is still a lack of information on the enzymatic behavior of these extracts and their specific action in the hydrolysis of buffalo milk caseins.

Artichoke flower extract (*Cynara cardunculus* var. *scolymus*) is an example of a coagulant that can be used to coagulate buffalo milk for cheese production. It belongs to the *Asteraceae* family and the *Cynara cardunculus* species, the same family and species as the thistle flower (*Cynara cardunculus* L. and *Cynara humillis* L*.)*. This plant has been used for many years as a milk coagulant in the production of traditional cheeses with protected designation of origin (PDO) in countries such as Portugal and Spain (Manuelian et al. 2020).

The artichoke flower is a perennial herbaceous plant native to southern Europe, in the Mediterranean region. Its consumption and cultivation increase every year, and it is commonly used as an ingredient in various culinary recipes. Furthermore, artichokes have been used as an alternative to calf rennet in cheese production, representing an excellent source of aspartic proteases (Ayuso et al. 2024). There is limited knowledge about the enzymatic action of artichoke flower extract on buffalo milk casein during coagulation, and there are not many studies that reveal its effectiveness as a coagulant for this milk.

Milk coagulation is the most crucial step in cheese production. The primary coagulant used is chymosin, generally obtained from microbial sources. This enzyme preferentially hydrolyzes the Phe105–Met106 peptide bond of κ‐casein (κ‐CN), destabilizing the micelles and forming a clot (Wiśniewski et al. 2025; Zhang et al. 2024). The hydrolysis of κ‐CN destabilizes, the micelles both electrostatically and sterically, triggering, in the presence of Ca^2+^, the micellar aggregation process (Salvador et al. 2022).

The hydrolysis process and the conditions under which it occurs vary among different classes of proteolytic enzymes, including cysteine, metalloproteases, serine proteases, and aspartic proteases. Aspartic proteases are more specific and function similarly to chymosin, producing firm gels. In contrast, cysteine and serine proteases are less selective, which can affect both texture and flavor. Metalloproteases tend to be more active but are less specific in their action. The origin of the enzyme (whether from animal, microbial, or plant sources) directly influences its kinetic behavior and thermal stability. Additionally, the species of milk used plays a crucial role in determining its composition, which impacts how the milk responds to the coagulant. This is particularly significant due to structural variations in the micelles found in cow, goat, sheep, and buffalo milk (de Farias et al. 2020; de Jesus et al. 2024).

To optimize enzyme kinetics, conditions such as temperature, pH, and salt (NaCl and CaCl_2_) concentrations are usually adjusted. Temperature is a critical factor in enzymatic reactions. It is essential to use a temperature range that ensures the stability of both enzymes and substrates (Wiśniewski et al. 2025).

The pH of the medium plays a crucial role in enzymatic hydrolysis. It influences the characteristics of the enzyme's functional groups, particularly those located at the active sites. This can alter the extent to which these functional groups associate or dissociate. The salt concentration will also affect the enzymatic reaction, the amount needs to be balanced for the enzyme to work well. The addition of electrolytes reduces the overall electrostatic repulsion between the molecules (Jesus et al. 2023).

Studying the behavior of enzymes extracted from artichoke flowers and their role in the hydrolysis of buffalo casein can lead to important information. Colombo et al. (2021) claim that as enzymatic milk‐clotting exerts a significant impact on the characteristics of the final product, this product should be carefully studied to determine the optimal conditions in the production of cheese from this coagulant. Given the previous information, the objective was to evaluate the behavior of proteases from artichoke flower extract during the enzymatic hydrolysis of buffalo casein and its technological application for cheese production.

## 2 Materials and Methods

### 2.1 Obtaining and Preparing Extracts

The artichoke flowers (*Cynara cardunculus* L. var *scolymus*) were purchased directly from producers in the São Roque region, São Paulo, Brazil (23° 31′ 45′ South 47° 08′ 07′ West) in the November 2022 harvest, and microbial chymosin (*Aspergillus niger* var *awamori*) (HÁ‐LA, Chr. Hansen, Brazil) was purchased from local stores to be used as a control, as it is the most widely used coagulant in the industry today.

The extracts were prepared according to the methodology of Jesus et al. (2023), with some adaptations. The pistils of the artichoke flowers were dried for 15 days at room temperature (±25°C) while being protected from light. After drying, 35 g of pistils were macerated in a mortar with 0.5 L of distilled water. The pistils were left to infuse for 15 min, then filtered through quantitative filter paper (Unifil, C40, 18,5 cm) and stored at –18°C for later analysis. Artichoke flower extract was prepared in three replicates.

### 2.2 Proteolytic Study of Buffalo Casein

#### 2.2.1 Obtaining Buffalo Casein

For the proteolytic analyses, buffalo milk casein was prepared according to the method described by Egito et al. (2006). Buffalo milk was collected from crossbred female buffaloes (Jafarabadi × Murrah) in the morning between January and February 2023. Initially, the milk was skimmed using centrifugation (MPW‐350, Warsaw, Poland) at 2100 × g for 30 min at 32°C. Total casein was then isolated by isoelectric precipitation, adjusting the milk's pH to 4.6 with 1 mol L^−1^ HCl. This mixture was centrifuged again (MPW‐350, Warsaw, Poland) at 1100 × g for 20 min at 32°C. The casein precipitate was solubilized in distilled water and its pH was gradually adjusted to 7.0 with small amounts of 1 mol/L NaOH, used only for neutralization and not in excess, in order to avoid the formation of sodium caseinate. The sample was then dialyzed using deionized water at 4°C. The casein solutions were lyophilized using a FreeZone 4.5 L (LabConco, Kansas City, MO, USA) and stored at –18°C until needed.

#### 2.2.2 Determination of Protein Contente

The protein content of the extract and microbial chymosin was determined by the methodology of Bradford (1976), using bovine serum albumin (BSA, Sigma‐Aldrich, St. Louis, MO, EUA) as default. Absorbances were measured in a spectrophotometer (Shimadzu UV‐1800, Duisburg, Germany) the 595 nm.

#### 2.2.3 PA

The PA of artichoke flower extract and microbial chymosin was determined using the method described by Mohanty et al. (2003) with adaptations. In this analysis, various conditions of pH, temperature, NaCl, and CaCl_2_ concentrations were utilized to observe the behavior of coagulant proteases under these conditions.

The PA method is based on the preparation of the buffalo casein substrate obtained previously, dissolving 1% (m/v) of the same in 10 mmol/L sodium phosphate buffer (pH 6.5). For the test, 1 mL of this substrate was incubated with 100 µL of the plant extract or microbial chymosin at 37°C in a thermostatic bath (Tecnal, model Te‐184, São Paulo, Brazil) for 30 min. To stop the reaction 4 mL of trichloroacetic acid (TCA, Sigma‐Aldrich, St. Louis, MO, EUA) at a concentration of 6.5% (m/v) was added, and the mixture was allowed to stand for 30 minutes at room temperature (25°C). Then the precipitated proteins were removed by centrifugation (centrifuge, MPW‐350, Warsaw, Poland) at 5000 × g for 20 min. For the blank, casein substrate was added after enzyme inactivation by TCA.

The protein content in the supernatant was measured at 280 nm in a spectrophotometer (Shimadzu UV‐1800, Duisburg, Germany). To evaluate PA, a standard curve was generated using L‐tyrosine (Sigma‐Aldrich, St. Louis, MO, USA) at concentrations of 0, 20, 40, 60, 80, and 100 µg/mL. The resulting standard curve was described by the equation *y* = 6.4133*x* + 0.1735, with a coefficient of determination (*R^2^*) of 0.989. A unit of enzyme activity (U) is defined as the amount of enzyme needed to produce 1 µmol of L‐tyrosine equivalent in 1 mL of reaction per minute (Equation 1). The specific activity was obtained by the ratio between the activity and the protein concentration of the extract, and was expressed in units of enzymatic activity per mg of protein.

(1) $$PA\;\left(U/mL\right)=\frac{\left(\mu \frac{\mathrm{mol}}{\mathrm{mL}}L\;-\;\mathrm{Tyrosine}\;\mathrm{equivalent}\;\times \;\mathrm{Dilution}\;\mathrm{factor}\right)}{\left(\mathrm{Volume}\;\mathrm{of}\;\mathrm{enzyme}\;\mathrm{used}\;\times \;\mathrm{Time}\;\left(\min\right)\right)}\;$$

#### 2.2.4 Effect of pH, Temperature, NaCl, and CaCl_2_ Concentration

The effect of pH on PA was assessed by incubating the extract with a 1% (m/v) buffalo casein substrate at various pH levels (5.8, 6.3, 6.8, and 7.3) using a 10 mmol/L sodium phosphate buffer. The temperature effect was performed at different incubation temperatures (30, 40, 50, 60 and 70°C). To evaluate the effect of the salt concentrations, 100 µL of the solutions were added to the casein substrate. The concentrations used were: NaCl (0, 500, 1000, 1500, 2000, 2500 mmol/L) and CaCl_2_ (0, 20, 60, 100, and 140 mmol/L) (Dinâmica Química Contemporânea Ltda., São Paulo, Brazil). CaCl_2_ was added to buffalo milk to ensure adequate availability of ionic calcium for the action of coagulating enzymes. The analysis was performed in accordance with item 2.2.3 with the variations described. After being analyzed, the supernatants obtained were stored at –18°C for subsequent chromatographic analysis.

#### 2.2.5 Reverse Phase High Performance Liquid Chromatography (RP‐HPLC)

RP‐HPLC was performed on an equipment Agilent (HP Agilent 1260 Infinitty II, Santa Clara—Ca, EUA) of the supernatants obtained in the PA, following the methodology of Ong et al. (2007), with modifications. A C18 column was used (Zorbax SB‐C18; 4.6 mm DI (inner diameter) × 250 mm, 5 mm particle size), coupled to a pre‐column (Zorbax SB‐C18, 4.6 mm ID × 12,5 mm, 5µm). Two mobile phases were utilized, with the first (A) consisting of a acetonitrile solution (5%, v/v) from (Dinâmica Química Contemporânea Ltda., São Paulo, Brazil) and trifluoroacetic acid (0.1%, v/v) (Dinâmica Química Contemporânea Ltda., São Paulo, Brazil) and the second phase (B) formed by acetonitrile (50%, v/v) and trifluoroacetic acid (0.1%, v/v).

A 20 µL sample of the supernatant was injected into the column at a flow rate of 1 mL/min using a gradient of mobile phases A and B over 70 min. The analysis began with a flow of 100% mobile phase A, which decreased linearly over 60 min to 100% mobile phase B. In the remaining time, a new linear gradient was established to return the flow to 100% mobile phase A, and this composition was maintained until the end of the analysis, which lasted a total of 70 min. Detection was carried out at 215 nm using a UV‐Vis detector. The retention time of the fractions, the peak area percentages, and their UV spectra were considered to evaluate the chromatograms.

### 2.3 Study of Buffalo Milk Coagulation

#### 2.3.1 MCA

The coagulating activity of artichoke flower extract and microbial chymosin was determined according to Bey et al. (2018), with modifications, and evaluated under different conditions of pH, temperature, and concentrations of NaCl and CaCl_2_.

The substrate used for this analysis was pasteurized buffalo fluid milk (64 ± 1°C/30 min). Before incubation, the milk was placed in a thermostatic bath (Tecnal, model Te‐184, São Paulo, Brazil) at 37°C for 10 min. The analysis involved adding 1 mL of buffalo milk to a test tube, followed by 10 µL of CaCl_2_ at 10 mmol/L to replace the soluble calcium lost during the pasteurization process, and finally, 100 µL of coagulant. The clotting time was measured by manually rotating the tube at short intervals and checking for visible clots on the tube wall. The time was recorded in seconds. For each assay, the clotting time of the sample was measured three times. MCA was expressed in Soxhlet units (SU), which represents the volume of milk that can be coagulated by a unit volume of the enzyme extract in 40 min at 37°C (Equation 2). The specific coagulation activity was obtained from the ratio between MCA and the protein concentration of the extract, and was expressed in Soxhlet units per mg of protein.

(2) $$MCA\;\;\left(SU/mL\right)=\frac{\left(2400\times V\right)}{\left(t\times v\right)}$$

*V*: is the volume of milk (mL); *v*: is the volume of enzyme (mL); *t*: is the coagulation time in seconds

#### 2.3.2 Effect of pH, Temperature, NaCl and CaCl_2_ Concentration

The effect of pH was analyzed by adjusting the pH of milk (approximately 6.8) with the addition of lactic acid (Dinâmica Química Contemporânea Ltda., São Paulo, Brazil), to decrease pH and sodium chloride 0.1 mol/L (Dinâmica Química Contemporânea Ltda., São Paulo, Brazil), to increase the pH, reaching treatments with pH values of 5.8, 6.3, 6.8, and 7.3, with the aid of a digital pH meter (model Q400AS, QUIMIS, Diadema, São Paulo, Brazil). The pH adjustment was performed as described by Bey et al. (2018), where sodium chloride is used to increase the pH of milk in order to reproduce the original experimental conditions and avoid significant chemical changes in the composition of milk that could occur with the use of strong bases.

The effect of temperature was determined by varying the incubation temperature (30, 40, 50, 60 and 70°C). The effect of NaCl (0, 500, 1000, 1500, 2000, 2500 mmol/L) and CaCl_2_ (0, 20, 60, 100, and 140 mmol/L) concentrations was performed using 10 µL of the solutions replacing the CaCl_2_ added in the MCA. The analysis was performed according to item 2.3.1 with the variations described.

The buffalo milk clots obtained were centrifuged (centrifuge, MPW‐350, Warsaw, Poland), filtered on quantitative filter paper (Unifil, C40, 18.5 cm) and stored at −18°C for later analysis.

#### 2.3.3 Sodium Dodecyl Sulfate‐Polyacrylamide Gel Electrophoresis (SDS‐PAGE)

Electrophoresis (SDS‐PAGE) of the clots obtained by MCA was performed in order to verify the difference in protein breakdown under the conditions used. The stacking gel concentration was 4% and the separating gel concentration was 12% according to Laemmil (1970). Aliquots of 10 µL of the samples were transferred to the gels, and the runs were performed at 4°C for 120 minutes at 250 V, 30 mA, and 15 W (Apelex PS 304 Minipac II, France). Molecular weight standards were used (Bio‐Rad, Hércules, CA, EUA) as a marker. After the run, the proteins were stained with Coomassie Blue (0.1%, m/v) (Dinâmica Química Contemporânea Ltda., São Paulo, Brazil) bleached with ethanol solution (30%, v/v) (Dinâmica Química Contemporânea Ltda., São Paulo, Brazil) and acetic acid (7.5%, v/v) (Dinâmica Química Contemporânea Ltda., São Paulo, Brazil), and the digitized gels. The molecular weights of the proteins migrated in the gel were determined according to Iizuka and Faust (1982).

#### 2.3.4 Mid‐Infrared Spectroscopy (MIR)

The clots were subjected to MIR analysis on a Fourier Transform Infrared Spectroscopy with Attenuated Total Reflection instrument (FTIR‐ATR) (AgilentCary 630 FTIR, Agilent Technologies Inc., Santa Clara, CA, USA), under the mid‐infrared range, with wavenumber from 4000 cm^−1^ to 600 cm^−1^. The spectra were obtained in absorbance mode, checking the crystal between each sample. The maximum absorbances associated with their wavenumber range were used as study variables for statistical analysis and subsequent comparison of their spectroscopic profile.

### 2.4 Technological Application

#### 2.4.1 Determination of SR

To evaluate the applicability of artichoke flower extract in the production of buffalo cheeses, the SR of coagulants was calculated under all pH and temperature conditions analyzed. The SR is the ratio between MCA and PA and was determined according to Equation 3. The best conditions obtained were used in the production of mini cheeses.

(3) $$SR=\frac{\mathrm{MCA}}{\mathrm{PA}}$$

#### 2.4.2 Mini Cheese Production

To study the viability of using artichoke flower extract in buffalo cheese production regarding yield, mini cheeses (approximately 15 g) were produced following the methodology of Mazorra‐Manzano et al. (2013) with adaptations. Two batches of mini cheeses were prepared: the first with artichoke flower extract and the second with microbial chymosin, each having three replicates. The pH, temperature, NaCl and CaCl_2_ concentration conditions with the best SR values were used in the processing. Coagulation was carried out by transferring 45 mL of buffalo milk into each centrifuge tube. Each tube was maintained at the selected temperature. To one tube, 1 mL of artichoke flower extract was added, while 0.5 mL of microbial chymosin was added to the other tube. The mixtures were allowed to coagulate for 60 min. After this period, the clot was manually cut with the aid of a stainless steel cutter and gently shaken by inverting the tubes 10 times and leaving to rest for 10 min. After resting, the samples were centrifuged at 1700 × g for 30 min (centrífuge, MPW‐350, Warsaw, Poland). The whey was collected and stored, the curd was molded into small cylindrical containers (5 cm in diameter) covered with gauze and stored for 6 h under refrigeration (4°C) for later analysis.

#### 2.4.3 Cheese Yield

The cheese yield was calculated according to Equation 4:

(4) $$Yield\;\%=\frac{\mathrm{Weight}\;\mathrm{of}\;\mathrm{curd}}{\mathrm{Weight}\;\mathrm{of}\;\mathrm{milk}}\;\;\times 100$$

#### 2.4.4 Cheese and Whey Analysis

Cheese and whey were analyzed for total dry extract (AOAC 926.08) and total protein by the Kjeldahl method (AOAC 2001.14) (AOAC 2016). The cheese samples were also analyzed for their electrophoretic profile in accordance with item 2.3.3 to observe the effect of the optimal conditions used on the milk proteins.

### 2.5 Experimental Design

The analyses were performed in a completely randomized design, in 3 replicates. To evaluate the PA and MCA, factorial schemes were performed, where the coagulant factor was used for all factorials, with 2 levels (artichoke flower extract and microbial chymosin). For the effect of temperature and CaCl_2_ concentration, 5 levels were used (factorial 2 × 5), for pH, 4 levels (factorial 2 × 4) and for NaCl concentration, 6 levels were used (factorial 2 × 6). Analysis of variance (ANOVA) was performed to evaluate the effect of treatments on PA and MCA. Regression was performed with a significance level of 5%. Tukey's mean test (*P* < 0.05) was performed on the SR data to verify the difference between the conditions analyzed.

Principal component analysis (PCA) was performed on the data obtained by RP‐HPLC, where peaks with the same retention time and their respective area percentages (%) were selected. These data were standardized with mean = 0 and standard deviation = 1. The number of principal components (PCs) was determined based on the interpretable factors and Kaiser criteria. These criteria select the initial PCs that represent more than 70% of the variance in the original data and have eigenvalues greater than 1, respectively. Statistical analyses were performed using the statistical program Statistical Analysis System (SAS) statistical software program, Student version 9.1 (Statistical Analysis System 2025).

## 3 Results and Discussion

### 3.1 Proteolytic Study of Artichoke Flower Extract on Buffalo casein

#### 3.1.1 Protein Content of Extract Effect of pH, Temperature, NaCl, and CaCl_2_ Concentration on PA

Artichoke flower extract and microbial chymosin were analyzed for their protein content as part of the characterization of the coagulants used. The artichoke flower extract had a protein content of 3.09 mg/mL, which was significantly higher (*p* < 0.05) than microbial chymosin, which showed a content of 0.60 mg/mL. These values are similar to those reported for the two coagulants by Jesus et al. (2023). This enzyme concentration directly affects coagulation properties, coagulant specificity and PA.

**FIGURE 1:**
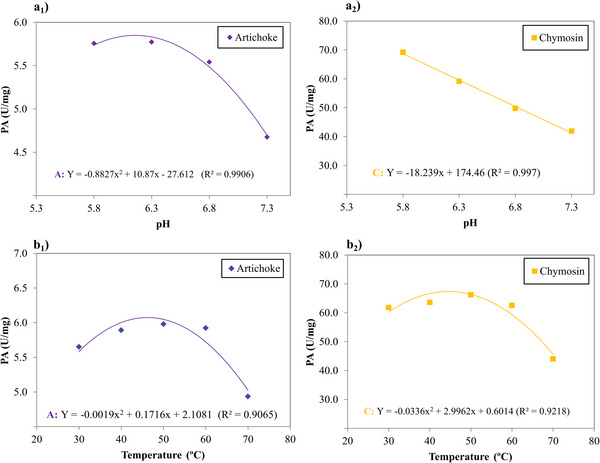
(a) Effect of pH changes on PA: (a_1_) artichoke flower extract; and (a_2_) microbial chymosin; (b) effect of temperature changes on PA: (b_1_) artichoke flower extract; and (b_2_) microbial chymosin. Regression model and *R^2^*.

The coagulants were tested for specific PA under varying conditions of temperature, pH, NaCl, and CaCl_2_ concentrations. Both pH and temperature significantly affected the results, allowing for the fitting of both linear and quadratic models (*p* < 0.05). Based on the correlation coefficient and the lack of adjustment (non‐significant), it was proven that the models were efficient in explaining the behavior of coagulants in the PA of the different conditions analyzed. The effect of the conditions used, the regression model adjusted for pH and temperature and the *R^2^* are described in Figure 1.

PA is crucial for evaluating an enzyme's effectiveness in milk coagulation. It is associated with proteolysis, impacting casein degradation and affecting both cheese yield and sensory properties. Every enzyme has an optimal temperature and pH for maximum activity, these factors directly influence their activation and inactivation. Plant proteases typically exhibit high PA, resulting in the production of smaller peptides in cheese. This process can lead to a bitter taste and alterations in texture (Luo et al. 2018).

In Figure 1, the measured activities showed higher values for microbial chymosin compared to the artichoke flower extract in all conditions analyzed. The pH had a linear decreasing effect in relation to the specific PA for the commercial microbial chymosin, indicating that as the pH of the medium increased, the PA value was reduced (Figure 1a_2_). Microbial chymosin showed maximum activity at pH 5.8. In contrast, artichoke flower extract, there was a quadratic behavior in relation to pH (Figure 1a_1_), presenting a maximum activity at pH 6.15. The maximum activity of artichoke flower extract at pH 6.15 is slightly higher than the pH range generally reported for aspartic proteases, which have optimal activity between pH 3 and 5.5. In this case, the crude extract may contain unpurified plant enzymes with optimal activity slightly shifted towards neutral pH. The study by Bueno‐Gavilá et al. (2020) showed that artichoke flower extract achieved its maximum PA between pH 5.5–6.2 under bovine casein.

The temperature showed quadratic behavior for both coagulants (Figure 1b), showing that PA tends to fall with increasing temperature. Microbial chymosin exhibited maximum activity of 67.40 U/mg at a temperature of 44.6°C, while artichoke flower extract demonstrated maximum activity of 5.98 U/mg at 45.2°C. Jesus et al. (2023) found an optimal temperature value of 48.6°C for artichoke flower extract in PA with bovine caseinclose to that found in the present study. The authors also noted that proteases may lose their activity at temperatures exceeding this optimal value.

Although microbial chymosin showed higher PA values compared to artichoke flower extract, both coagulants exhibited similar behaviors with temperature variations, demonstrating slight increases in activity with rising temperatures. However, beyond 60°C, this activity began to decline. Although temperatures above 60°C are not used in cheese processing, they were included to evaluate the thermal behavior of enzymes and their stability. Evaluation over a wide temperature range is essential to determine the denaturation point and possible loss of enzymatic activity, which is relevant information for technological applications and for understanding the thermal profile of the plant extract.

The use of calcium and sodium plays an important role in cheese production, having a direct influence on the structure of casein, enhancing the effect of proteases and increasing the coagulation rate. In this study, the concentrations of NaCl and CaCl_2_ used did not significantly impact the PA values (*p* > 0.05), indicating no variation in PA at the tested concentrations. This result may be associated with the low concentrations of salts used, which were probably insufficient to modify the ionic strength of the medium or affect the conformation of the proteases present in the artichoke flower extract and microbial chymosin.

#### 3.1.2 RP‐HPLC

**FIGURE 2:**
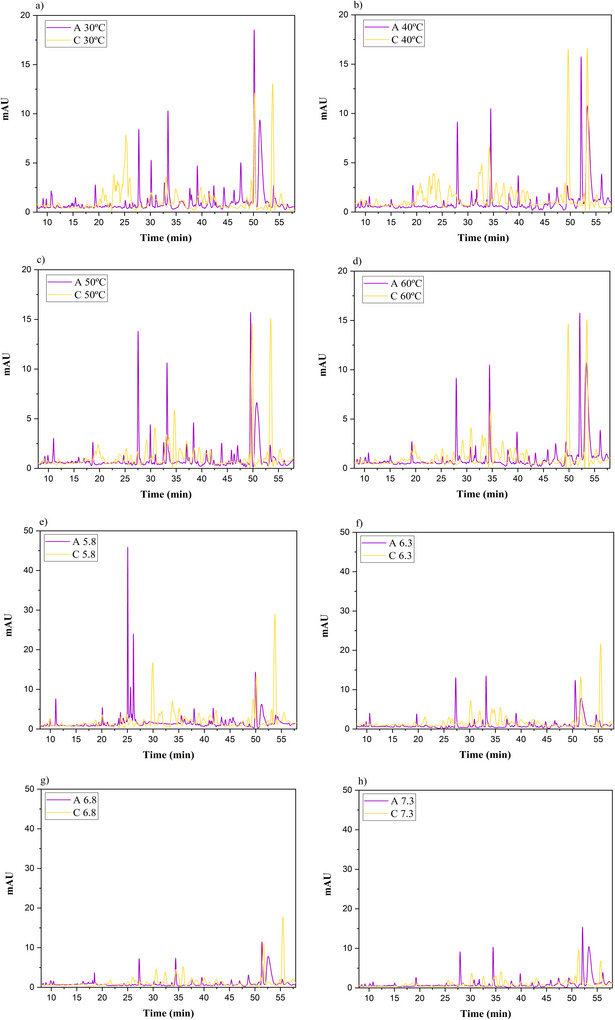
Chromatograms representing the profile of TCA soluble peptides hydrolyzed with microbial chymosin (C) and artichoke flower extract (A), obtained by RP‐HPLC under different temperature and pH conditions: (a) temperature of 30°C; (b) temperature of 40°C; (c) temperature of 50°C; (d) temperature of 60°C; (e) pH 5.8; (f) pH 6.3; (g) pH 6.8; and (h) pH 7.3.

After performing the proteolytic analysis, the supernatants from (pH and temperature) treatments were collected and analyzed by RP‐HPLC to evaluate the similarities and differences in the peptide samples generated from buffalo casein. The peptide profile of the pH variation samples showed 77 peaks of the same UV spectrum and 55 peaks for the temperature treatment. The representative chromatograms (Figure 2) show differences in the profile of TCA soluble peptides obtained under different pH and temperature conditions. The number and intensity of the peaks observed between 5 and 25 min indicate the presence of lower molecular weight peptides, while peaks retained after 25 min suggest more hydrophobic and larger peptides.

PCA was performed on the RP‐HPLC data. Initially, the entire dataset, which encompassed the full chromatogram, was used, with each peak area (%) considered a variable. The correlation between the variables and the PCs was analyzed, along with the sum of squares of the PCs. Variables with the lowest correlation and the smallest sum of squares were gradually excluded until the most significant variables were identified.

**FIGURE 3:**
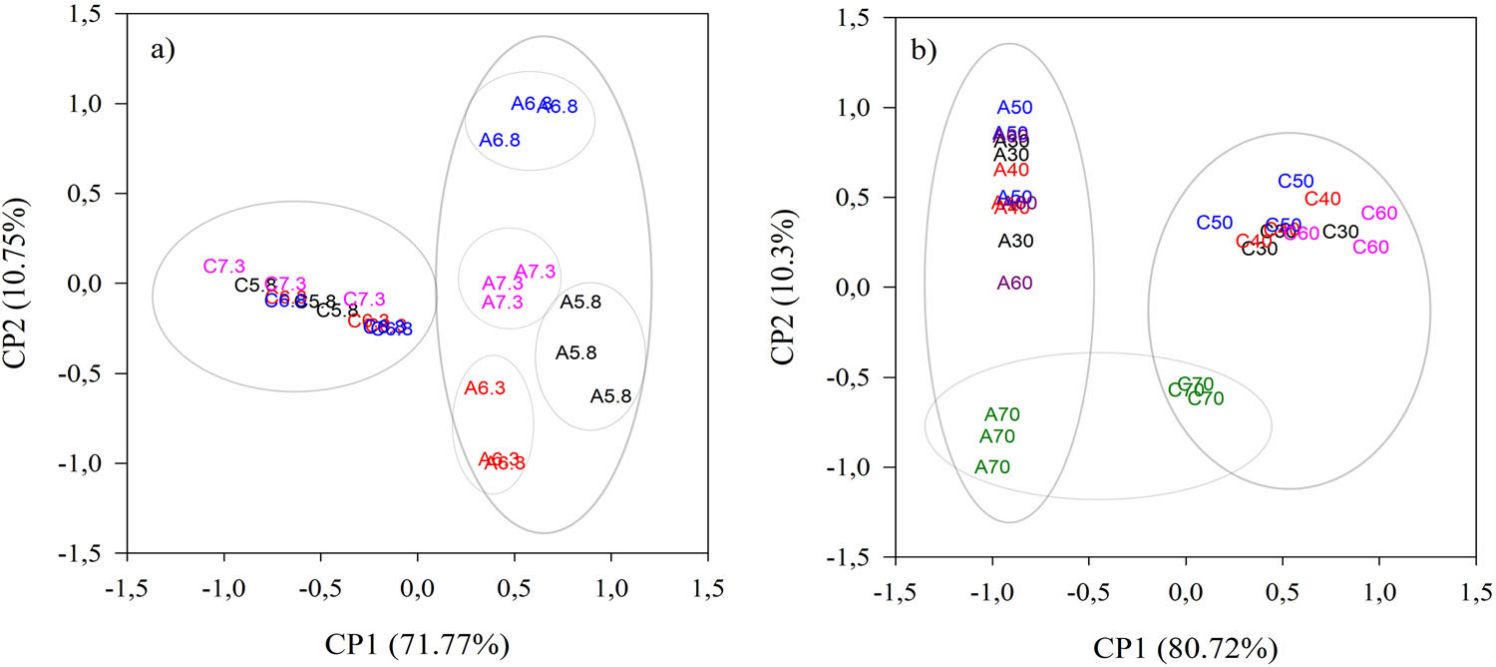
Scatter plot of the main components of the peptide fractions obtained by RP‐HPLC in the PA samples for the coagulants: Microbial chymosin (C) and Artichoke flower extract (A); (a) effect on pH change: 5.8 (A5.8 and C5.8), 6.3 (A6.3 and C6.3), 6.8 (A6.8 and C6.8) and 7.3 (A7.3 and C7.3); (b) temperature: 30°C (A30 and C30), 40°C (A40 and C40), 50°C (A50 and C50) and 60°C (A60 and C60).

Seventeen peaks were selected for the samples with pH variation and twenty‐one peaks for temperature, these being the most significant and best reflecting the variance of the original data. These selected variables were described by principal componente 1 (PC1) and principal component (PC2) for the pH and temperature conditions (Figure 3).

The PCA results indicated differences in the proteolytic action of coagulants on buffalo casein, leading to the separation of samples hydrolyzed with artichoke flower extract and microbial chymosin.

In the pH variation, the data distribution was best represented by PC1 (71.77%), where the samples were separated according to the type of coagulant used in the PA (Figure 3a). The samples treated with microbial chymosin showed a lower percentage of the eluted peptides at retention times of 25.05 and 26.84 min. These peptides are derived from the degradation of β‐casein (β‐CN) and α_S1_‐casein, which can be observed at retention times ranging from 23 to 40 min (Sulejmani et al. 2020). From the PCA, it is also possible to verify that the variation in pH for microbial chymosin did not present a difference in the composition of the peptides.

PC2 (10.75%) played a significant role in promoting the separation of sample groups using artichoke flower extract based on their pH values. This component is positively correlated with peak 63, observed at 43.54 min. The samples positioned on the positive side of PC2 exhibit a higher percentage of area for this peak, indicating a greater proteolytic effect of artichoke flower extract on buffalo casein. This result indicates that while both coagulants affected PA, only the artichoke flower extract demonstrated a change in the peptide profile resulting from the hydrolysis of buffalo casein at different pH levels.

Figure 3b illustrates the dispersion of the samples under various temperature conditions. The PC1 accounts for 80.72% of the variation in peptide content among the samples. This component is correlated with the peaks 21, 27, and 48, indicating that the samples obtained with the artichoke flower extract (located on the negative side of PC1) exhibited a higher percentage of area for these peaks. For the two coagulants used, it was not possible to observe any difference in the peptide composition in relation to the variation in room temperature, except for the temperature of 70°C. This separation was promoted by PC2 (10.3%), which correlated negatively with peaks 9, 16, 43, and 45, indicating that the increase in temperature may have resulted in the denaturation of the enzymes, which may have influenced the low formation of peptides at this temperature.

While artichoke flower extract has a different enzymatic action than microbial chymosin, resulting in a distinct peptide profile, it exhibits similar thermal stability to chymosin. High temperatures can impact the enzymatic activity of both types of coagulants.

The results indicate that environmental conditions can influence the enzymatic activity of coagulants, leading to the formation of different peptides from the hydrolysis of buffalo casein. These peptides will directly affect the characteristics of the cheese produced, potentially being either desirable or undesirable. In addition to PA, it is important to study the action of the enzyme on milk proteins, in order to evaluate MCA and thus determine the most important parameter, the SR.

### 3.2 Study of Buffalo Milk Coagulation

#### 3.2.1 Effect of pH, Temperature, NaCl, and CaCl_2_ Concentration on MCA

**FIGURE 4:**
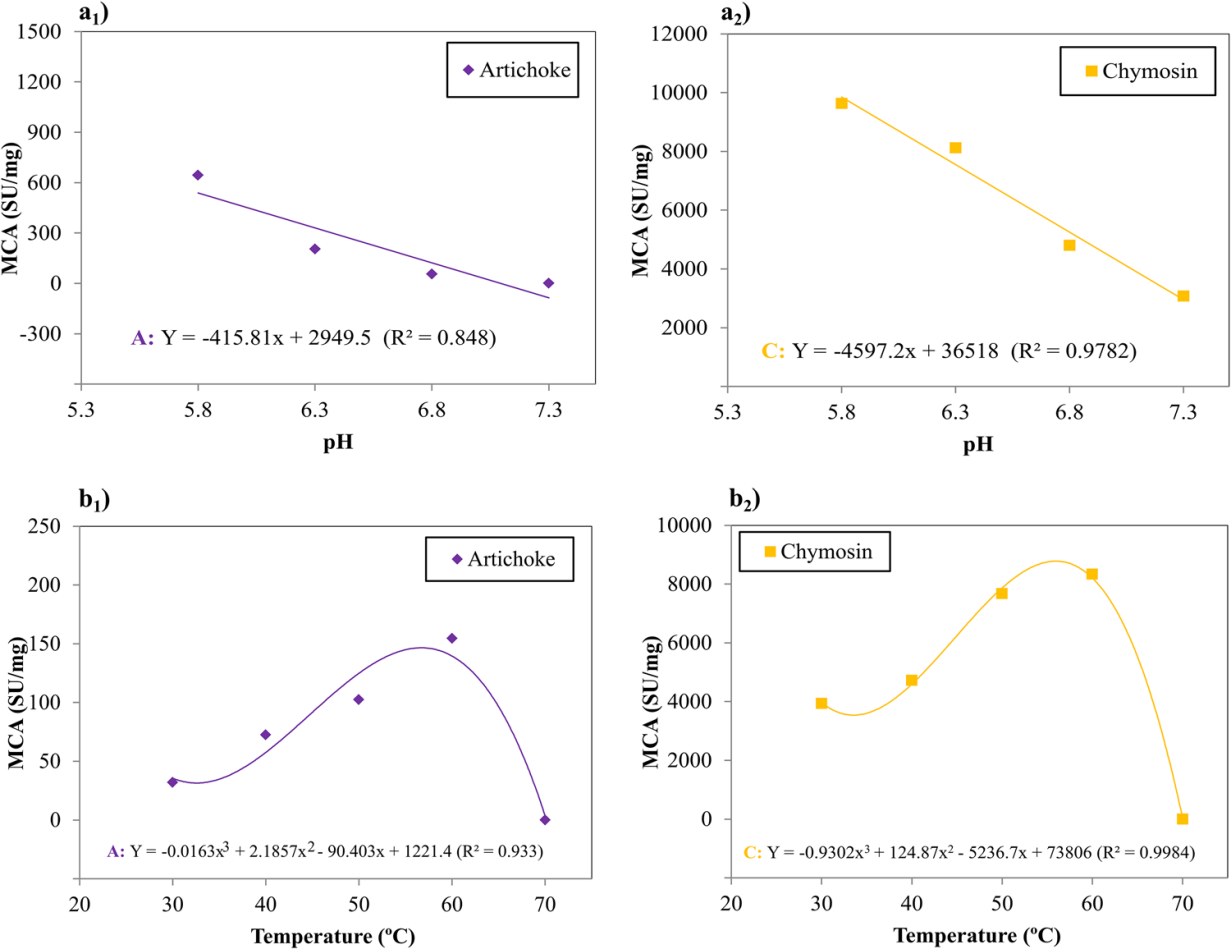
(a) Effect of pH changes on MCA: (a_1_) artichoke flower extract; and (a_2_) microbial chymosin; (b) effect of temperature changes on MCA: (b_1_) artichoke flower extract; and (b_2_) microbial chymosin. Regression model and *R^2^*.

Both pH and temperature significantly influenced the coagulation activity of buffalo milk. This allowed the development of linear and polynomial models (*p* < 0.05) that adequately explained the behavior of specific milk coagulation activity (MCA) under various conditions. The analysis of lack of fit yielded a non‐significant result, confirming the suitability of the models. Figure 4 illustrates the impact of pH and temperature on MCA, along with the regression model and the coefficient of determination (R^2^).

Milk coagulation is the most important step in cheese production and can be affected by factors such as pH, temperature, salt concentration, milk composition, among others. In MCA, the enzymes used also have optimal pH and temperature levels that enhance their effectiveness on casein (Bey et al. 2018).

In all variations tested in the MCA, microbial chymosin and artichoke flower extract demonstrated a statistically significant difference (*p* < 0.05) within each treatment. Microbial chymosin likely had a higher MCA value because it is a commercial enzyme produced on an industrial scale and has undergone a purification process, whereas the artichoke flower extract used was crude.

In Figure 4a, it is possible to observe that both microbial chymosin and artichoke flower extract showed a decreasing linear effect on MCA as milk pH increased. Both coagulants showed maximum coagulation activity at pH 5.8. At a pH close to 5.5, the ionic strength of the medium is influenced, leading to significant changes in the coagulation process. A decrease in the pH of the milk reduces charge repulsion and increases the ionic concentration of calcium. As a result, the casein micelles tend to move closer together (Britten and Giroux 2022).

There was no coagulation of buffalo milk at pH 7.3 with artichoke flower extract (zero activity). These PA and MCA results confirm that plant extracts and microbial chymosin have greater activity at more acidic pH (5.5–6.1). The pH of buffalo milk is approximately 6.8 and to achieve better activity of these enzymes in the production of buffalo cheese, there is the alternative of acidifying the medium by adding a dairy culture during the pre‐maturation stage.

The two coagulants presented third‐order polynomial models with temperature variation (Figure 4b), showing that the enzymes presented an optimal temperature for maximum coagulation, but as this temperature increased, coagulation activity decreased. Microbial chymosin presented maximum activity at a temperature of 55.9°C, and artichoke flower extract presented maximum activity at a temperature of 56.9°C, values close to those found in PA. The maximum activity observed at high temperatures may be related to the greater thermal stability of recombinant chymosin (Ahmed et al. 2016) and the presence of multiple proteases in artichoke flower extract with different ranges of thermal stability (Chazarra et al. 2007). At 70°C, there was no coagulation for any of the coagulants.

Chazarra et al. (2007) explain that the decline in coagulation activity is due to the loss of enzyme stability, which alters its active sites. This decline is attributed to the denaturation of serum proteins, leading to the formation of complexes involving k‐casein, β‐lactoglobulin, and α‐lactalbumin. In cheese production, high temperatures can lead to fragile curds and reduced production yields, resulting in losses for the industry. Therefore, lower coagulation temperatures are utilized.

Similar to PA, the coagulants did not demonstrate a significant effect (*p* > 0.05) on the studied NaCl and CaCl_2_ concentrations, with no statistical difference in MCA values. This can be explained by the high association of calcium with micelles in buffalo milk. About 78–80% of total calcium is in colloidal form (associated with colloidal calcium phosphate), a higher proportion than that observed in cow's milk (approximately 65%), which confers greater colloidal stability and lower sensitivity to small concentrations of salts (Mejares et al. 2023). In addition, due to the higher calcium content in buffalo milk (180 mg/100 mL) and the relatively low ionic fraction (20–22%), the addition of CaCl_2_ is generally not necessary in the coagulation of buffalo cheeses (Deshmukh et al. 2024).

#### 3.2.2 Characterization of Clots

Based on the differences obtained from the artichoke flower extract in the MCA and PA analyses for pH and temperature variations, the behavior of buffalo milk under these conditions was studied through characterization by electrophoretic and spectroscopic analysis. For these analyses, the clots obtained in the MCA of the pH and temperature variation were used in order to better understand the observed differences. Since there was no coagulation at pH 7.3 and at a temperature of 70°C, these samples were not analyzed further.

##### 3.2.2.1 Electrophoresis(SDS‐PAGE)

SDS‐PAGE electrophoresis is commonly used to examine casein hydrolysis and proteolysis rates in cheeses. Casein fractions from cheese samples were identified by comparing the relative mobilities of their proteins to those of standard proteins.

Figure 4 displays the protein profiles of coagulated buffalo milk under various pH and temperature conditions. The identified proteins include α_s1_‐casein (α_s1_‐CN), α_s2_‐casein (α_s2_‐CN), β‐CN, and κ‐CN. The whey proteins such as α‐lactalbumin and β‐lactoglobulin, which are trapped in the clot matrices, were also identified (Gonçalves et al. 2017; Mbye et al. 2023).

**FIGURE 5:**
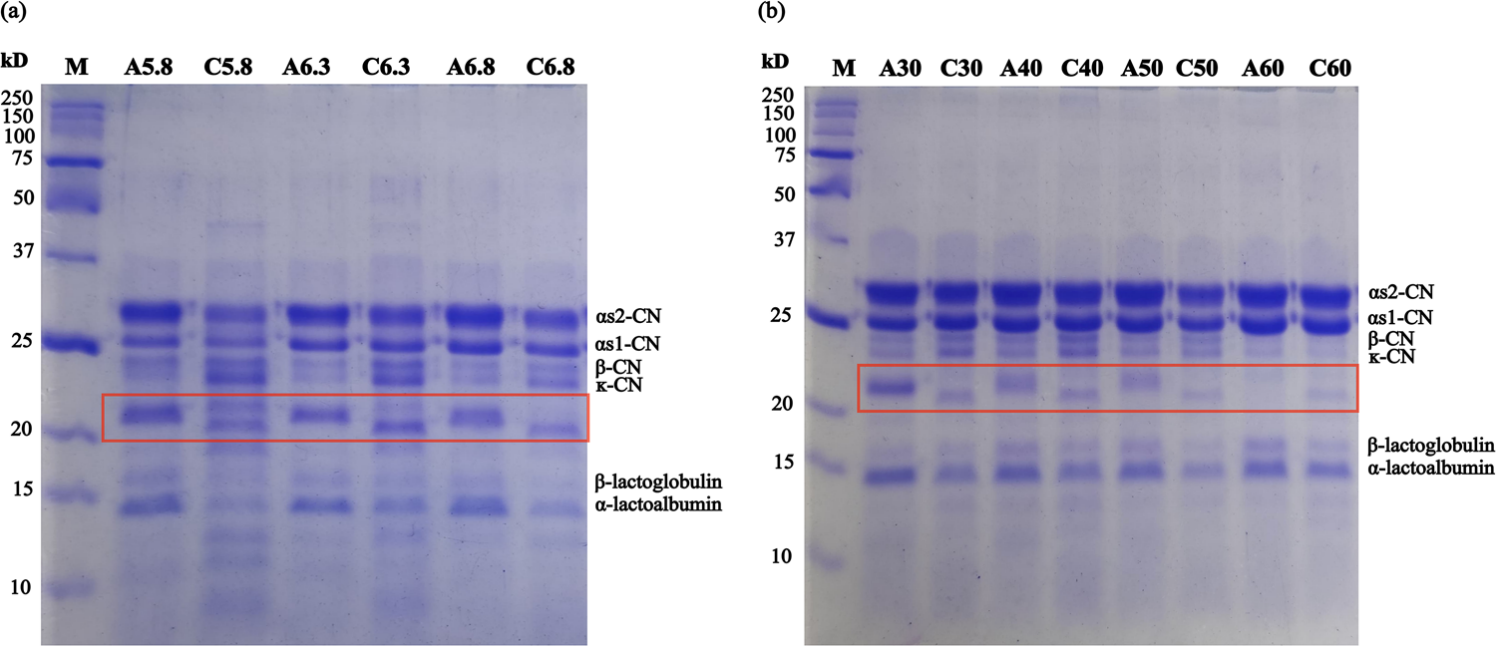
Representative SDS‐PAGE gel of buffalo milk clots coagulated with artichoke flower extract (A) and microbial chymosin (C); (a) different pH conditions: 5.8 (A5.8 and C5.8), 6.3 (A6.3 and C6.3), 6.8 (A6.8 and C6.8) and 7.3 (A7.3 and C7.3); (b) different temperature conditions: 30°C (A30 and C30), 40°C (A40 and C40), 50°C (A50 and C50) and 60°C (A60 and C60).

For the variation in pH and coagulation temperature, no differences were observed between samples of the same coagulant. A difference was observed between the coagulants, regardless of the conditions used, in a protein fraction generated in the samples (highlighted in red). In the pH variation (Figure 5a), the samples containing artichoke flower extract showed a molecular weight of approximately 21.57 kDa, while the samples with chymosin exhibited a molecular weight of about 20.47 kDa.

In relation to the temperature variation (Figure 5b), the samples coagulated with artichoke flower extract showed a molecular weight of 22.12 kDa for the corresponding fraction, while chymosin had a molecular weight of 20.60 kDa. In the sample coagulated at a temperature of 60°C using artichoke, this fraction was not detected.

This fraction can be attributed to the hydrolysis of β‐CN, which results from secondary proteolysis. The stability of β‐CN can be compromised by several factors, including the type of heat treatment applied, the duration of acidification in the dough due to the use of lactic cultures (which can vary in type, quantity, and concentration), and the hydrolysis of κ‐CN promoted by the action of rennet (Gonçalves et al. 2017).

The proteases being studied (A and C) exhibited similar activity in hydrolyzing k‐casein, specifically breaking the Phe105‐Met106 bond of κ‐CN and converting it into para‐κ‐CN. This hydrolysis removes the hydrophilic glycomacropeptide segment, which exposes hydrophobic regions that facilitate micellar aggregation in the presence of calcium ions (Walstra and Almudi 2001). In contrast, the artichoke extract demonstrated greater PA, in addition to hydrolyzing the specific linkage of k‐casein, it also hydrolyzed β‐CN at various linkages when compared to microbial chymosin, resulting in peptides of different molecular weights.

A reduction in the k‐casein band was observed when using artichoke flower extract, both in response to changes in pH and variations in temperature. The formation of para‐κ‐CN is indicated by the decrease in the intensity of the κ‐CN band (approximately 19 kDa) seen in all gels, particularly in those that contained artichoke flower extract.

Timón et al. (2019) state that enzymes from different sources, whether animal, plant, or microbial, vary in their specificity and thus influence the primary proteolysis of casein.

##### 3.2.2.2 MIR

**FIGURE 6:**
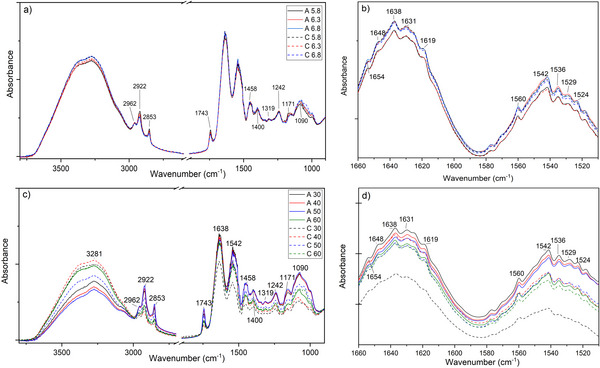
Spectra of buffalo milk clots coagulated with artichoke extract (A) and microbial chymosin (C) obtained by MIR: spectrum of the 4000 – 900 cm^−1^ region of the samples with different pH conditions: 5.8 (A5.8 e C5.8), 6.3 (A6.3 e C6.3) e 6.8 (A6.8 e C6.8); (b) enlarged region of 1660 – 1510 cm^−1^ of the different pH; (c) spectrum of the 4000 – 900 cm^−1^ region of the samples with different temperature conditions: 30°C (A30 e C30), 40°C (A40 e C40), 50°C (A50 e C50) e 60°C (A60 e C60); and (d) enlarged region of 1660 – 1510 cm^−1^ of different temperature conditions.

Infrared analysis revealed significant differences in the coagulation mechanisms among the coagulants (Figure 6). In the spectra found in the 1700–1500 cm^−1^ region, which is associated with proteins, two important bands stood out: Amide I and Amide II. The Amide I band exhibits the most intense absorption and is linked to the stretching vibrations of the carbonyl group (C ═ O), along with CN stretching and NH angular deformation, occurring within the 1700–1600 cm^−1^ range. This band is particularly sensitive to changes in the secondary structure of proteins, such as α‐helices and β‐sheets. In contrast, the Amide II band corresponds to CN stretching and NH angular deformation, observed in the 1600–1500 cm^−1^ range. The Amide I band is the most sensitive to structural changes, due to hydrogen bonding and coupling between transition dipoles (Ye et al. 2017).

The analysis highlighted the different proteolytic mechanisms employed by the coagulants. At pH 5.8, the stronger intensity of the bands associated with microbial chymosin indicates a more selective hydrolysis of κ‐CN, which helps maintain the secondary structure of the other caseins. In contrast, the weaker intensity of the bands observed with the artichoke flower extract suggests a more extensive and non‐specific proteolysis, resulting in greater overall protein hydrolysis.

The differences in conformation are especially noticeable in the changes to the Amide I band (1638 cm^−1^), which indicate the structural rearrangement of caseins during coagulation. The artichoke flower extract induces more significant modifications in the secondary structure, suggesting a distinct mechanism of action compared to chymosin, which preserves the proteins' structural integrity to a greater extent.

The main finding regarding the different temperatures tested is that the artichoke flower extract exhibits greater thermal sensitivity. This is evidenced by the progressive reduction in the intensity of the Amide I (1638 cm^−1^) and II bands (1542 cm^−1^) as the temperature increases, which indicates a more pronounced denaturation of proteins. In contrast, microbial chymosin demonstrates greater conformational stability, exhibiting more consistent spectral patterns across the varying temperatures.

Both coagulants promote curd formation, however, artichoke flower extract induces more significant structural changes in buffalo milk proteins, particularly under high‐temperature conditions. These changes can affect both the yield and sensory quality of the cheese produced, as high PA may lead to the formation of hydrophobic peptides, which can contribute to a bitter taste. According to de Jesus et al. (2024), cheeses made with artichoke flower extract were noted for their bitter aftertaste. Despite this characteristic, consumers still enjoy the cheese (Barracosa et al. 2021).

### 3.3 Technological Application

Microbial chymosin is considered the best coagulant due to its specificity in breaking down κ‐CN between the amino acids Phe_105_–Met_106_ resulting in two fractions, offering the best yields during cheese making. When studying plant extracts as milk coagulants and possible substitutes for microbial chymosin, it is essential to evaluate their specificity and yield in curd formation (Mazorra‐Manzano et al. 2013). The SR of the analyzed conditions was calculated to determine the optimal conditions for buffalo cheese production and to evaluate the curd yield.

#### 3.3.1 Determination of SR

The SR is an index used to evaluate the suitability of a coagulant for cheese production. It is calculated by the ratio between the MCA and PA values.

The results indicated that the artichoke flower extract showed higher values at pH 5.8 (388.2) and a temperature of 60°C (90.7) (Table S1). High temperatures during the cheese production process can lead to the denaturation of proteins, as noted by Singh et al. (2016). This denaturation is typically undesirable because it changes the physical and chemical properties of the proteins, resulting in reduced solubility and functionality, which ultimately leads to a decrease in yield. A higher energy expenditure would be necessary for this production. Therefore, the chosen temperature for calculating the yield was 40°C (42.9). This temperature corresponds to the values of the coagulants that were closest to each other and is also the temperature most commonly used in the majority of dairy products.

#### 3.3.2 Electrophoresis (SDS‐PAGE)

**FIGURE 7:**
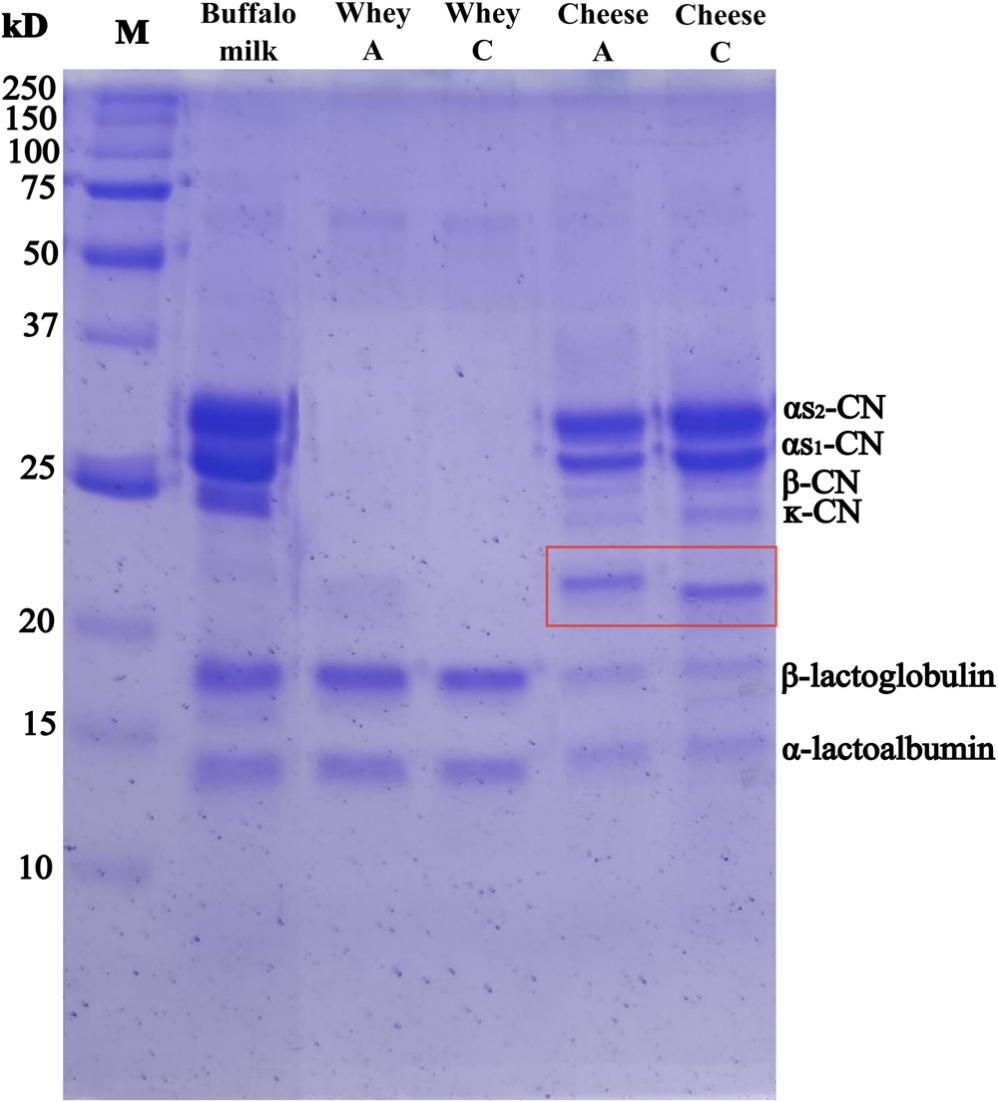
Representative SDS‐PAGE gel of whey and cheese samples from buffalo cheese coagulated with artichoke flower extract (A) and microbial chymosin (C); (M) molecular weight marker.

To verify the activity of the enzymes found in the artichoke flower extract under the selected production conditions, we conducted an electrophoresis analysis (SDS‐PAGE) on samples of buffalo milk, whey, and mini cheese produced with the artichoke flower extract, as well as whey and mini cheese made with microbial chymosin (Figure 7).

The presence of α_s1_, α_s2_, β, κ‐CN, α‐lactalbumin, and β‐lactoglobulin was observed in milk and cheese samples that were coagulated using artichoke flower extract and microbial chymosin. In the serum samples, α‐lactalbumin and β‐lactoglobulin were also detected, with no differences found in these fractions among the samples.

In the cheese samples, a small difference was observed in the protein fractions around 20 kDa. The cheese produced with artichoke flower extract showed a fraction with a higher molecular weight compared to the cheese made with microbial chymosin. A difference was also observed in the intensity of the α_s1_ and κ‐CN bands in the cheese samples. The cheese made with microbial chymosin exhibited a greater intensity of these bands, indicating a higher concentration of these protein fractions in that sample. This difference is associated with the specificity of microbial chymosin in the hydrolysis of κ‐CN, releasing the caseinomacropeptide and efficiently destabilizing the casein micelles, preserving part of the κ‐CN in the gel formed. Conversely, plant coagulants exhibit less specificity and can degrade κ‐CN as well as other proteins, such as αs‐CN (Timón et al. 2019).

The results suggest that despite the use of optimal conditions for the enzymatic activity of the extract, it can probably still hydrolyze buffalo milk proteins at other active sites.

#### 3.3.3 Production and Yield of Buffalo Milk Cheese

Table 1 describes the mean values and standard deviations of the yield of mini cheeses, dry extract and protein content of the cheese and whey, obtained during production.

**TABLE 1:** Mean values and standard deviations of yield, dry extract and cheese protein, dry extract and whey protein, obtained with artichoke flower extract and microbial chymosin.

Parameters (%)	Coagulants	
	Artichoke flower extract	Microbial chymosin
Cheese yield	33.44 ± 1.38 ^a^	35.02 ± 1.09 ^a^
Cheese dry extract	28.57 ± 0.68 ^a^	27.68 ± 0.97 ^a^
Whey dry extract	7.44 ± 1.06 ^a^	8.63 ± 1.73 ^a^
Cheese protein	19.00 ± 1.51^a^	18.85 ± 0,43 ^a^
Whey protein	1.27 ± 0.04 ^a^	1.11 ± 0.03 ^b^

*Note*: ^a,b^ Results followed by the same letter in the row do not differ (*p* < 0.05) in the F test.

All parameters evaluated showed a significant difference (*p* > 0.05) between the artichoke flower extract and microbial chymosin only regarding the protein content of the obtained serums. These results are of great importance, as they show that even though the artichoke flower extract presents lower coagulation values than buffalo milk in relation to microbial chymosin, its use did not present a difference in yield.

The artichoke flower extract exhibited a higher protein content in the collected serum, likely due to the high specificity of microbial chymosin. Despite the difference in serum protein levels, the overall protein content of the cheese did not show a significant difference (*p* < 0.05) between the coagulants. This indicates that although microbial chymosin is highly specific and the artichoke flower extract may produce smaller peptides that could be lost in the serum, the cheese made with artichoke flower extract maintained its protein content.

The yield of artichoke flower extract in coagulating buffalo milk is satisfactory, as the results showed no significant difference compared to the industry‐standard coagulant, microbial chymosin. The cheeses produced with artichoke flower extract had similar protein content, dry extract levels, and overall yield. Artichoke flower extract can be considered a good buffalo milk coagulant for cheese production, which can add value to the products, encouraging buffalo cheese producers to invest in new products with differentiated characteristics.

## 4 Conclusion

The artichoke flower extract displayed optimal conditions specific PA at pH 6.15 and 45.2°C, while microbial chymosin was best at pH 5.8 and 44.6°C. RP‐HPLC confirmed differences in peptide fractions from buffalo casein hydrolysis for all studied conditions. Both coagulants for specific milk coagulation showed maximum activity at pH 5.8, with artichoke flower extract at 56.9°C and microbial chymosin at 55.9°C. SDS‐PAGE and RP‐HPLC analyses revealed that casein was broken into different peptides by artichoke flower extract compared to chymosin, which was also confirmed by MIR spectroscopic analysis. The artichoke flower extract effectively hydrolyzed casein at different bonds without affecting the protein content or yield of buffalo cheese, suggesting it is a promising coagulant for producing value‐added products.

## Nomenclature

PA: proteolytic activity
MCA: milk clotting activity
RP‐HPLC: Reverse Phase High Performance Liquid Chromatography
SDS‐PAGE: Sodium Dodecyl Sulfate‐Polyacrylamide Gel Electrophoresis
MIR: Mid‐Infrared Spectroscopy
TCA: trichloroacetic acid
SR: specificity ratio
PCA: Principal component analysis
A: artichoke flower extract
C: microbial chymosin.

## Author Contributions

**Rebeca Rodrigues Vieira Onelli**: investigation, writing – original draft, methodology, data curation, project administration, conceptualization. **Josane Cardim de Jesus**: conceptualization, methodology. **Thinara de Freitas Oliveira**: methodology. **Leduan Rosa Alcântara**: methodology. **Renata Cristina Ferreira Bonomo**: writing – review and editing. **Leandro Soares Santos**: writing – review and editing, methodology. **Sibelli Passini Barbosa Ferrão**: supervision, conceptualization, writing – review and editing.

## Conflicts of Interest

The authors declare no conflicts of interest.

## Supporting information




**Supplementary Materials**: jfds70773‐Sup‐0001‐SuppMat.docx
