# Supplementary material for: Behavior of Aspartic Proteases From Artichoke Flower (Cynara cardunculus L. var Scolymus) in the Hydrolysis of Buffalo Milk Casein
Source: J Food Sci. 2025 Dec 15;90(12):e70773. doi: 10.1111/1750-3841.70773 (PMC12703569; doi:10.1111/1750-3841.70773)
Supplement: Supplementary file 1 — Supplementary Materials: jfds70773‐Sup‐0001‐SuppMat.docx [file JFDS-90-0-s001.docx]

**Supplementary material**

Table S1. Mean values ​​and standard deviations of RE for pH and temperature conditions using artichoke extract (A) and chymosin (C).

| **RE** | **pH** | | | | | | | |  |
| --- | --- | --- | --- | --- | --- | --- | --- | --- | --- |
|  | 5.8 | | 6.3 | | 6.8 | | 7.3 | |  |
| **A** | 388.2 ± 12.68^a^ | | 123.2 ± 7.24^b^ | | 35.3 ± 2.11^c^ | | 0 ± 0.00^d^ | |  |
| **C** | 85.1 ± 6.95^a^ | | 85.1 ± 11.22^a^ | | 57.7 ± 2.95^b^ | | 46.4 ± 4.16^b^ | |  |
|  | **Temperature (°C)** | | | | | | | |  |
|  | 30 | 40 | | 50 | | 60 | | 70 | |
| **A** | 19.7 ± 1.70^a^ | 42.9 ± 3.42^b^ | | 51.5 ± 2.97^c^ | | 90.7 ± 5.66^d^ | | 0 ± 0.00^e^ | |
| **C** | 38.5 ± 1.66^a^ | 44.8 ± 4.28^a^ | | 70.0 ± 8.42^b^ | | 80.4 ± 5.02^b^ | | 0 ± 0.00^c^ | |

^a,b^ Results followed by the same letter in line do not differ (P > 0.05) by the Tukey test.
